# Inclusion of the severe and enduring anorexia nervosa phenotype in genetics research: a scoping review

**DOI:** 10.1186/s40337-024-01009-9

**Published:** 2024-04-29

**Authors:** Sarah Ramsay, Kendra Allison, Heide S. Temples, Luigi Boccuto, Sara M. Sarasua

**Affiliations:** 1 Healthcare Genetics and Genomics Program, School of Nursing, Clemson University, Clemson, SC 29634, USA; 2 School of Nursing, Clemson University , Clemson, SC 29634, USA

**Keywords:** Severe enduring anorexia nervosa, Genetics, Review

## Abstract

**Background:**

Anorexia nervosa has one of the highest mortality rates of all mental illnesses. For those who survive, less than 70% fully recover, with many going on to develop a more severe and enduring phenotype. Research now suggests that genetics plays a role in the development and persistence of anorexia nervosa. Inclusion of participants with more severe and enduring illness in genetics studies of anorexia nervosa is critical.

**Objective:**

The primary goal of this review was to assess the inclusion of participants meeting the criteria for the severe enduring anorexia nervosa phenotype in genetics research by (1) identifying the most widely used defining criteria for severe enduring anorexia nervosa and (2) performing a review of the genetics literature to assess the inclusion of participants meeting the identified criteria.

**Methods:**

Searches of the genetics literature from 2012 to 2023 were performed in the PubMed, PsycINFO, and Web of Science databases. Publications were selected per the Preferred Reporting Items for Systematic Reviews and Meta-Analyses extension for Scoping Reviews (PRISMA-ScR). The criteria used to define the severe and enduring anorexia nervosa phenotype were derived by how often they were used in the literature since 2017. The publications identified through the literature search were then assessed for inclusion of participants meeting these criteria.

**Results:**

most prevalent criteria used to define severe enduring anorexia nervosa in the literature were an illness duration of ≥ 7 years, lack of positive response to at least two previous evidence-based treatments, a body mass index meeting the Diagnostic and Statistical Manual of Mental Disorders-5 for extreme anorexia nervosa, and an assessment of psychological and/or behavioral severity indicating a significant impact on quality of life. There was a lack of consistent identification and inclusion of those meeting the criteria for severe enduring anorexia nervosa in the genetics literature.

**Discussion:**

This lack of consistent identification and inclusion of patients with severe enduring anorexia nervosa in genetics research has the potential to hamper the isolation of risk loci and the development of new, more effective treatment options for patients with anorexia nervosa.

**Supplementary Information:**

The online version contains supplementary material available at 10.1186/s40337-024-01009-9.

## Abstract (plain language summary)

Anorexia nervosa (AN) is a serious illness with a high death rate. Many of those with AN do not recover and have continuing severe psychological and physical symptoms that greatly impact their quality of life. Research has shown that genetics plays an important role, along with environment, in the development and persistence of AN. This review highlights the continued lack of consensus on defining criteria for severe and enduring AN in the literature and the continued focus on younger females with shorter illness durations in AN genetics research. Greater efforts are needed to include older participants with severe AN of longer duration in genetics research in hopes of developing more effective treatments for this underrepresented group.

**Supplementary Information:**

The online version contains supplementary material available at 10.1186/s40337-024-01009-9.

## Background

Anorexia nervosa (AN) is a devastating illness with a high mortality rate. The standardized mortality ratio (SMR) calculates whether those in a given study population are equally, more or less likely to die compared to a reference population [1]. With an estimated SMR between 5.9 and 15.9 (i.e., 6–16 times excess mortality), AN is considered one of the deadliest mental disorders [2, 3].

Studies indicate that the overall incidence rate for AN has remained relatively stable (4% female lifetime-0.3% male lifetime) since the 1970s [2, 4]. The symptomology and presentation of AN have evolved along cultural lines; however, it is not simply a manifestation of modern cultural and social pressures. Accounts of deliberate self-starvation date back to the beginning of written history [5].

Although the exact etiology of AN is still unclear, a substantial body of evidence indicates that genetics plays a considerable role [6, 7]. Genetic studies dating from the late 20th century have shown that AN is highly familial. The lifetime risk of developing AN for female relatives of individuals with AN is 11 times greater than that for female relatives of individuals without AN [8]. Heritability (h^2^_twin_) estimates from twin studies range from ∼48–74% [9–16]. The large range in estimates may be due to the use of broader participant inclusion criteria in AN studies to increase study group size. Broadening the inclusion criteria results in a more heterogeneous sample and decreased heritability estimates, while narrowing the definition of AN yields higher and more consistent estimates [17].

Although recovery from AN is possible, for approximately 20% of affected individuals the condition takes on a more intractable phenotype [18, 19]. While AN symptoms vary from person to person, it has been suggested that a unique severe and enduring anorexia nervosa (SE-AN) subtype exists; however, aligning on clear defining criteria has proved challenging [20].

Since the 1980s, a small number of literature reviews of varying breadth and depth have been conducted in attempts to better define SE-AN. The most comprehensive to date, a 2017 review by Broomfield and colleagues identified illness duration and previous unsuccessful treatment as the criteria most often used in the literature to define AN severity [21]. A 2018 editorial by Hay and Touz, which referenced the Broomfield review, expanded the suggested criterion to include significantly diminished quality of life and narrowed the duration criterion to a minimum of three years and the therapeutic intervention exposure criterion to at least two previous evidence-based treatments [22]. In a 2021 follow-up review, with the aim of defining a neuropsychological profile for SE-AN, Bloomfield et al. identified intelligence, set-shifting and decision-making as features warranting further attention and noted that additional data are needed to align on defining severity criteria [23]. In short, there continues to be a lack of consensus on how to best define SE-AN.

Psychiatric illness is often diagnosed in a binary manner; an individual is assessed as either having the illness or not. In reality, due to their complex nature, psychiatric illnesses are better defined on a continuum [24, 25]. Genome-wide association studies (GWAS) often use a binary case-control design. However, as Yang et al. [26] noted, with an equal population sample size, a quantitative trait (for example, symptom severity) association study will have greater power than a case-control association study. The difference is because in a case-control study, an individual with mild symptoms is not differentiated from one with severe symptoms. Relating this to AN, there would be no differentiation between an individual who met the DSM-5 criteria for mild illness, of short duration and who was responsive to first-line treatment, and an individual who met the extreme illness criteria, with a duration of over a decade and lack of positive response to multiple treatment modalities. Delineating participants based on illness severity when performing genetic data analysis of those with AN may improve the chances of identifying significant variants.

The potential value of defining more phenotypically similar groups based on quantitative phenotypes and comorbidities in genetic studies of psychiatric illness has been demonstrated in major depressive disorder (MDD), schizophrenia, autism spectrum disorder (ASD), and obsessive-compulsive disorder (OCD) [27–30]. Individuals with more severe MDD symptoms have been found to have increased genetic risk for other psychiatric disorders [29], and polygenic risk scores (PRS) for schizophrenia correlate with symptom severity [28]. Genetic risk score (GRS), PRS and polygenic score (PGS) are the terms most often used in the literature when referring to values estimating an individual’s lifetime risk of developing a phenotype (disorder) based only on their genetics [31]. The scores are generated by combining the number of risk alleles at all the risk variants in an individual’s genome. Disease-associated risk variants are based on the latest and most comprehensive GWAS for the disorder at the time of the analysis.

Studies delineating and comparing subgroups of individuals with AN based on defined quantitative criteria may result in the discovery of rare variants associated with symptom severity, and individuals manifesting a more severe phenotype may be more likely to show higher heritability estimates and thus represent a subgroup of patients for which genetics findings may be beneficial. However, this hypothesis cannot be adequately tested to the rigorous standards required without a more precise definition of what constitutes a severe and enduring phenotype, and greater attention given to specifically identifying and including this group in genetic studies [32].

The aim of this review is to first, as an extension of the Broomfield et al. review [21], identify the criteria most widely used to describe the phenotypic severity of AN by including articles published since 2017 and, second, evaluate the genetics literature for inclusion of individuals meeting these criteria.

## Methods

### Delineating criteria for the severe and enduring anorexia nervosa phenotype

To better identify and delineate research participants manifesting a severe and enduring phenotype in the genetics literature, it was necessary to discern the most often used defining criteria for this subgroup of AN. The terms Anorexia Nervosa AND severe AND (Enduring OR Chronic) were used, with no year limit, to search titles and abstracts in PubMed, PsycINFO, and Web of Science. Articles were also limited to human subjects.

One of the articles identified was an extensive review by Broomfield et al. of how the literature labeled and defined AN severity up to 2017 [21]. The current search was limited to articles published after the Broomfield 2017 review to focus on the most recent literature. The references were not required to be attempting to empirically define a severe or enduring anorexia nervosa phenotype. The goal was to determine how those with a longer lasting and more severe clinical presentation are currently referred to in the literature. After removing commentaries on other references, clarifications, and updates from previous studies with the same authors and criteria, redundant references, and those not referring to a severe or enduring anorexia nervosa phenotype, 37 publications remained. Of these 37 publications, there were 22 research papers (6 clinical trials, 16 studies), 4 case reports, 6 expert panel/position papers/or opinion/editorial papers, 2 literature reviews and 3 general reviews. These references are listed in Table 1, along with a book chapter [33] identified through reviewing the references of the selected papers, that was not included in the Broomfield 2017 review, bringing the total publications included to 38. The mean age, mean BMI, duration of illness in years, and history of previous treatment, as well as any other measures of illness severity, were extracted from the articles and are shown in Table 1. A second reviewer, using the RANBETWEEN function in Microsoft Excel, selected 10% of the articles at random from Table 1. to review for meeting inclusion criteria and accuracy of the data extracted.

**Table 1:** Articles for Assessment of Severe Enduring Anorexia Nervosa (SE-AN) criteria

Reference		Overview	Title	Mean BMI kg/m^2^ (SD)	Duration years	Previous treatment history	Other Measure of Severity
1.	[36] Ambwani et al., 2020	Retrospective study- Patients grouped into early stage and SE-AN	A multicenter audit of outpatient care for adult anorexia nervosa: Symptom trajectory, service use, and evidence in support of “early stage” versus “severe and enduring” classification.	Of SE-AN group:15.95 (1.27)	Defined for SE-AN Group ≥ 7	Patients in the SE-AN group had been hospitalized more often than those in the early-stage group	Defined for SE-AN group: Severity of psychological distress (DASS-total ≥ 60)
2.	[62] Bemer et al., 2021	Retrospective study-BMD in “extremely malnourished” “extremely severe” female inpatients with AN	Bone mineral density at extremely low weight in patients with anorexia nervosa	Excluded if BMI ≥ 16 Of participants: 12.60 ± 1.60	Of participants:11.10 ± 10.40	Not reported	Assessed BMD but did not use this to define severity of AN.
3.	[58] Bianchi et al., 2021	Position paper presenting “harm reduction” approach in SE-AN	The Ethical Defensibility of Harm Reduction and Eating Disorders	Refers to hypothetical patient with SE-AN as having a BMI of 14	Definition: ≥6	Not reported for defining SE-AN	Not reported for defining SE-AN. Emphasis on improving quality of life.
4.	[21] Broomfield et al., 2017	PRISMA literature review with the goal of better defining criteria for SE-AN.	Labeling and defining severe and enduring anorexia nervosa: A systematic review and critical analysis	Range in included studies:13.31–19.80	Range in included studies:2.88–20.4	Studies including criteria for previous failed treatment attempt(s)	Measures indicative of significant impairment of quality of life, psychological and/or social.
5.	[34] Broomfield et al., 2021	Expert panel study to better define “later stage AN”	Establishing consensus for labeling and defining the later stage of anorexia nervosa: A Delphi study	Participants consensus that duration of illness, quality of life and unsuccessful attempts at evidence-based treatment are important to include in a universal definition of illness			
6.	[115] Calabrese et al., 2022	Clinical trial evaluating effectiveness of ketamine and a ketogenic diet on weight recovered participants with persistent eating disorder psychopathy.	Ketogenic diet and ketamine infusion treatment to target chronic persistent eating disorder psychopathology in anorexia nervosa: a pilot study	All considered “weight restored”	Inclusion criteria > 10	Not reported	All considered “weight restored” but with ongoing severe AN-related preoccupations
7.	[43] Calugi et al., 2017	Study evaluating outcomes of those with SE-AN and those with AN, but not SE-AN	Intensive enhanced cognitive behavioural therapy for severe and enduring anorexia nervosa: A longitudinal outcome study	Defined Severe (BMI 15-15.99) Defined Extreme (BMI < 15) For SE-AN group: 15.2 (2.0)	Definition for SE-AN group > 7 Participants in SE-AN group 12.3 (4.7)	84.4% of SEAN group noted recent treatment failure	EDE, EDE-12, GSI
8.	[45] Dalton et al., 2022	Clinical trial of rTMS effectiveness for SE-AN	My dad was like “it’s your brain, what are you doing?“’: Participant experiences of repetitive transcranial magnetic stimulation treatment in severe enduring anorexia nervosa.	Inclusion criteria > 14	Defining Inclusion criteria: ≥3 Participants: 15.05 ±11.33	Defining Inclusion criteria: ≥ 1 NICE-2017 recommended day or inpatient treatment. Participants: Number of previous hospitalizations 2.31±1.95 Mean duration of previous hospital stays in months 11.56 ±12.02	Various tools used though not to define SE-AN
9.	[46] Dalton et al., 2018	Clinical trial of rTMS effectiveness for SE-AN	Randomised controlled feasibility trial of real versus sham repetitive transcranial magnetic stimulation treatment in adults with severe and enduring anorexia nervosa: the TIARA study	Inclusion criteria > 14 Participants: 16 (1.44)	Defining Inclusion criteria: ≥3 Participants: 14.07 (10.75)	Defining Inclusion criteria: ≥ 1 NICE-2017 recommended day or inpatient treatment. Participants: Number of previous hospitalizations 2.18 (1.91) Mean duration of previous hospital stays in months 10.49 (11.66)	Various tools used though not to define SE-AN
10.	[47] Dalton, Foerde, et al., 2020	Clinical trial of rTMS effectiveness for SE-AN	The effect of repetitive transcranial magnetic stimulation on food choice-related self-control in patients with severe, enduring anorexia nervosa.	Inclusion criteria > 14 Participants: 15.90 ± 1.40	Defining Inclusion criteria: ≥3	Defining Inclusion criteria: ≥ 1 NICE-2017 recommended day or inpatient treatment.	Various tools used, though not to define SE-AN
11.	[48] Dalton, Lewis, et al., 2020	Clinical trial of rTMS effectiveness for SE-AN (follow-up)	Repetitive transcranial magnetic stimulation treatment in severe, enduring anorexia nervosa: An open longer-term follow-up	See Dalton 2018	See Dalton 2018	See Dalton 2018	See Dalton 2018
12.	[37] Fernandes Arroteia et al., 2020	Case report “severe” “chronic” AN response to Deep brain stimulation	Impressive weight gain after deep brain stimulation of nucleus accumbens in treatment-resistant bulimic anorexia nervosa	Participant: 12.8	Participant:26	“The patient has participated in various psychiatric therapies, including behavioural therapy”	Various tools used, though not to define SE-AN
13.	[116] Forbes, 2020	Opinion piece on withdrawal of care for adolescents with “severe anorexia”	Futility in adolescent anorexia nervosa and the question of withdrawal of care	Not included	Refers to > 7, though points out this cannot be applied for adolescents.	Not included	Not included
14.	[54] González Macías et al., 2021	Case report of intervention for SE-AN.	Group family psychotherapy during relapse. Case report of a novel intervention for severe and enduring anorexia nervosa	Participant: 16.0 No defining criteria for SEAN used	Participant:11 No defining criteria for SEAN used though “chronicity” noted as defining	Participant: None specifically for eating disorders No defining criteria for SEAN used though “treatment failure” noted as defining	Participant: Vomiting of 70 a week, compulsive exercise, self-injuries, risk behaviors
15.	[117] Guinhut et al., 2021	Study evaluating mortality for patient admitted to a specialized clinical nutrition unit for “extremely severe malnutrition”	Five-year mortality of severely malnourished patients with chronic anorexia nervosa admitted to a medical unit	Defining: <13 Participants: 12.7 (± 2.2)	Participants:9.8 ± 9.3	Participants: 2.9 ± 3.4	Various tools used though not to define SE-AN
16.	[22] Hay & Touyz, 2018	Editorial on defining SEAN	Classification challenges in the field of eating disorders: can severe and enduring anorexia nervosa be better defined?	Defining criteria: “underweight”	Notes that long duration is one criterion for SE-AN Agreed lower limit 3	Defining criteria: “Exposure to at least two evidence-based treatments appropriately delivered together with (an appropriate) diagnostic assessment”	Defining criteria: “A persistent state of dietary restriction, and overvaluation of weight/shape with functional impairment”
17.	[55] Hemmingsen et al., 2020	Case report of cognitive performance patient with “chronic” AN and “extremely low BMI”	Case report: cognitive performance in an extreme case of anorexia nervosa with a body mass index of 7.7	Participant: 7.2	Patient: 25	Patient: Repeated hospitalizations in treatment facilities specializing in eating disorders.	Various tools used though not to define SE-AN
18.	[57] Herpertz-Dahlmann, 2020	Commentary on detecting risk of developing “serious and enduring” AN	Serious and enduring anorexia nervosa from a developmental point of view: How to detect potential risks at an early stage and prevent chronic illness?	Not reported	Refers to Hay & Touyz, 2018: >3	Refers to Hay & Touyz et al., 2018: Exposure to at least two evidence-based treatments	Not reported
19.	[118] Israely et al., 2017	Clinical trial evaluating effectiveness of tyrosine in “severe hospitalized” AN patients	A Double-Blind, Randomized Cross-Over Trial of Tyrosine Treatment on Cognitive Function and Psychological Parameters in Severe Hospitalized Anorexia Nervosa Patients	Participants: 15.5 (1.6)	Participants: 6.3 (4.9)	Participants: Number of prior hospitalizations 1.4 (0.7)	Various tools used, though not to define SE-AN
20.	[50] Johansson et al., 2022	Study evaluating polygenic risk association with severity and outcome for “severe and enduring eating disorders”	Polygenic association with severity and long-term outcome in eating disorder cases	Participants: 15.91 (1.39) AN-R Participants: 16.52 (1.49) AN-BP	Follow-up time (difference in years between year at first registration and year when recontacted for recruitment) ≥ 5 years	Treatment resistance noted as important and as such all participants had undergone at least one treatment attempt	SEED definition: CIA score ≥ 18 and a follow-up time ≥ 5 years (i.e., years between initial registration and ANGI recruitment)
21.	[49] Knyahnytska et al., 2019	Study evaluating feasibility and safety of insula dTMS in those with SE-AN	Insula H-coil deep transcranial magnetic stimulation in severe and enduring anorexia nervosa (SEAN): A pilot study	Participants: 16.6 ± 0.9	Definition of treatment resistance: >5	Definition of treatment resistance: Failure to respond to at least 2 trials of intensive treatment interventions	Various tools used, though not to define SE-AN
22.	[59] Marzola et al., 2021	Study attempting to determine profiles for those with AN of varying duration. Those with LD-AN, have more previous unsuccessful treatment attempts, and lower lifetime BMI. Did not impact treatment response.	Inpatients with severe- enduring anorexia nervosa: Understanding the “enduringness” specifier	Participant in long duration group: At baseline 14.3 (2.1) Lowest lifetime 12.8 (1.9)	Long duration defined as ≥ 7	Participant in long duration group: Number of previous hospitalizations 2.6 (3.2) SD	Numerous assessments were conducted but none allowing for a clear definition of SEAN from non-SEAN AN
23.	[119] Mishima et al., 2021	Study using MRI to evaluate structural brain changes in those with SE-AN.	Structural brain changes in severe and enduring anorexia nervosa: A multimodal magnetic resonance imaging study of gray matter volume, cortical thickness, and white matter integrity.	Participants: 14.2 (2.5)	Participants: 15.7 (9.0)	Not reported	Various tools used, though not to define SE-AN
24.	[51] Oudijn et al., 2021	Study evaluating DBS in those with “treatment-refractory anorexia nervosa”	Deep brain stimulation of the ventral anterior limb of the capsula interna in patients with treatment-refractory anorexia nervosa.	Inclusion criteria: <15	Inclusion criteria: ≥10	Inclusion criteria: “Lack of response to ≥2 typical modes of treatment including ≥1 inpatient treatment of hospitalization”	Inclusion criteria: GAF score ≤45 for ≥2 years
25.	[64] Park et al., 2018	Study evaluating feasibility and efficacy of DBS in those with SE-AN	Study Protocol: Using Deep- Brain Stimulation, Multimodal Neuroimaging and Neuroethics to Understand and Treat Severe Enduring Anorexia Nervosa	Inclusion criteria: >13, < 16	Inclusion criteria: >7	Inclusion criteria: “lack of response to two or more typical modes of treatment, such as inpatient weight restoration, psychotherapy and psychopharmacology”	Inclusion criteria: Disabling severity with substantial functional impairment^1^
26.	[52] Pérez et al., 2022	Study evaluating efficacy of DBS in those with SE-AN.	Cognitive and quality-of-life related factors of body mass index (BMI) improvement after deep brain stimulation in the subcallosal cingulate and nucleus accumbens in treatment-refractory chronic anorexia nervosa	Inclusion criteria: >13, < 15.99 (participants outside this range could participate if meeting other criteria)	Inclusion criteria: ≥10	Inclusion criteria: Lack of response to ≥3 voluntary intensive treatments or clinical worsening and unwillingness to receive further treatment, including hospital admissions for involuntary feeding.	Various tools used, though not to define SE-AN
27.	[35] Raykos et al., 2018	Study examining whether AN duration or severity impacts response to cognitive behavioral therapy. Study found that they did not.	Severe and enduring anorexia nervosa? Illness severity and duration are unrelated to outcomes from cognitive behaviour therapy.	Participants: 16.8 (1.5)	Participants:5 [3 low, 12 high] Grouped into 3-, 7- and 11-year illness duration for assessment.	Participants: 91.8% and 55.2% had a history of psychological treatment and psychiatric hospitalizations, respectively.	Noted “the severity of problematic eating disorder attitudes and cognitions at pretreatment (had) no impact on the amount of change in BMI during the early phase of treatment.”
28.	[120] Russell et al., 2019	Literature review on “harm reduction” approach in SE-AN	Harm minimization in severe and enduring anorexia nervosa.	Does not propose criteria	Does not propose criteria	Does not propose criteria	Does not propose criteria
29.	[53] Scaife et al., 2022	Study evaluating efficacy of DBS in those with SE-AN	Deep brain stimulation of the nucleus accumbens in severe enduring anorexia nervosa: A pilot study.	Inclusion criteria:>13, < 16	Inclusion criteria:>7	Inclusion criteria: ≥3 voluntary intensive treatments (partial or full hospitalization) and ≥2 trials of psychological treatment	Various tools used though not to define SE-AN
30.	[70] Smith & Woodside, 2020	Retrospective study of data from inpatient eating disorder treatment facility to compare characteristics of patients with multiple admissions to those with good outcomes	Characterizing Treatment-Resistant Anorexia Nervosa	Participants in treatment- resistant group: 14.36 [13.56 low, 15.60 high]	Participants in treatment-resistant group: 6.84 [low 2.62, 12.32 high]	Treatment-resistant defined as two or more incomplete inpatient admissions and no complete admissions	Participants in treatment- resistant group: EDEQ 5.29 [low 4.76, high 5.59] and BDI: 46.00 [low 34.00, high 53.00]
31.	[121] Strand et al., 2020	Review paper providing background on palliative care for SE-AN.	A palliative care approach in psychiatry: Clinical implications	“More or less permanent low BMI”	“Longstanding Illness”	“Numerous treatment episodes have not resulted in any lasting remission”	“Patient who has ‘tried everything’ without success”
32.	[33] Sun et al., 2015	Chapter reviewing neurosurgical treatments for psychiatric conditions, including SEAN	Surgical treatments for anorexia nervosa	Participants weight must be < 85% of ideal body weight (and/or BMI < 17.5)	DSM-IV criterion C, and GAF ≤45 or less for at least 2 years	Participants must have been treated with an appropriate therapy for more than 3 years. At least two types of therapy must have been applied with no response.	“Must have experienced a rapid decrease in body weight over a short time period, which could be life-threatening without effective intervention”
33.	[122] Tumba et al., 2023	Perspective paper presenting the difficulties of SEAN cases, the harm reduction approach and physician-assisted suicide.	Clinical and Ethical Dilemmas in the Involuntary Treatment of Anorexia Nervosa	Definition: 13–14	Definition: 6–12	Not included	Definition: “Have decreased likelihood of remission, have acquired various physical consequences of prolonged disordered eating, potassium (3.0 mmol/L); persistent risk of acute medical decompensation and need for hospitalization.”
34.	[44] Voderholzer et al., 2018	Case study of “chronically ill” participant with “extreme” AN	3-year course after successful therapy of extreme anorexia nervosa	Participant: 8.5	Participant: 7	Participant: 3 prior inpatient stays in eating disorder specialty clinics	Participant: Extreme urge to move
35.	[56] Westermair et al., 2020	Perspective paper presenting palliative care for SE-AN	A palliative approach for severest anorexia nervosa?	Criteria for palliative care: Extremely low BMI (e.g. <13 kg/m^2^	Criteria for palliative care long duration of illness (e.g. 10 years)	Criteria for palliative care: High number of inpatient admissions and coercive measures without sustained symptom reduction (e.g.,>3)	Criteria for palliative care: Pronounced purging behavior. Psychiatric comorbidities Somatic comorbidities Unwillingness to change, identification with/pride in the disease
36.	[60] Wildes et al., 2017	Study on developing an empirically based definition of SE-AN	Characterizing severe and enduring anorexia nervosa: An empirical approach	Noted: “patients with AN could be grouped into higher and lower severity profiles that differed with respect to eating disorder behaviors and quality-of-life, as well as several external validators including age, weight concerns, and lifetime mood and anxiety comorbidity. Moreover, within each severity profile, there was dimensional variability in eating disorder behavior, quality-of-life, and indicators of chronicity (i.e., illness duration, repeated bouts of hospital treatment, and low BMI)”.			
37.	[20] (Wonderlich et al., 2020	Review of historical issues in diagnosing SE-AN as well as novel treatments and clinical perspectives	Severe and enduring anorexia nervosa: Update and observations about the current clinical reality.	Refers to Hays 2018 proposed criteria: “underweight”	Refers to Hay 2018 proposed criteria: >3	Refers to Hay & Touyz, 2018 proposed criteria: “Exposure to at least two evidence-based treatments appropriately delivered together with (an appropriate) diagnostic assessment”	Refers to Hay & Touyz, 2018 proposed criteria: “A persistent state of dietary restriction, and overvaluation of weight/shape with functional impairment”
38.	[69] Zhu et al., 2020	Review of psychological treatments for those with SE-AN	Psychological treatments for people with severe and enduring anorexia nervosa: A mini review	Refers to Hay 2018 proposed criteria: “underweight”	Refers to Hay & Touyz, 2018 proposed criteria: >3	Refers to Hay & Touyz, 2018 proposed criteria: “Exposure to at least two evidence-based treatments appropriately delivered together with (an appropriate) diagnostic assessment”	Refers to Hay & Touyz, 2018 proposed criteria: “A persistent state of dietary restriction, and overvaluation of weight/shape with functional impairment”

**AN-R**- Anorexia Nervosa-Restricting type; **AN-BP**- Anorexia Nervosa Binge Purge type; **BDI**-Beck Depression Inventory; **BMD-**Bone Mineral density; **BMI**-Body Mass Index; **CIA**- Clinical Impairment Scale; **DASS**-Depression, Anxiety, and Stress Scales; **EDE**- Eating Disorder Examination; **GAF**-Global Assessment of Function; **GSI**- Global Severity Index; **MRI**: Magnetic resonance imaging; **NA**-Not Applicable (and/or provided); **NICE-**National Institute for Health and Care Excellence; **rTMS**- Repetitive Transcranial Magnetic Stimulation; **SE-AN**- Severe and Enduring Anorexia Nervosa; **SD-**Standard Deviation

**Fig. 1:**
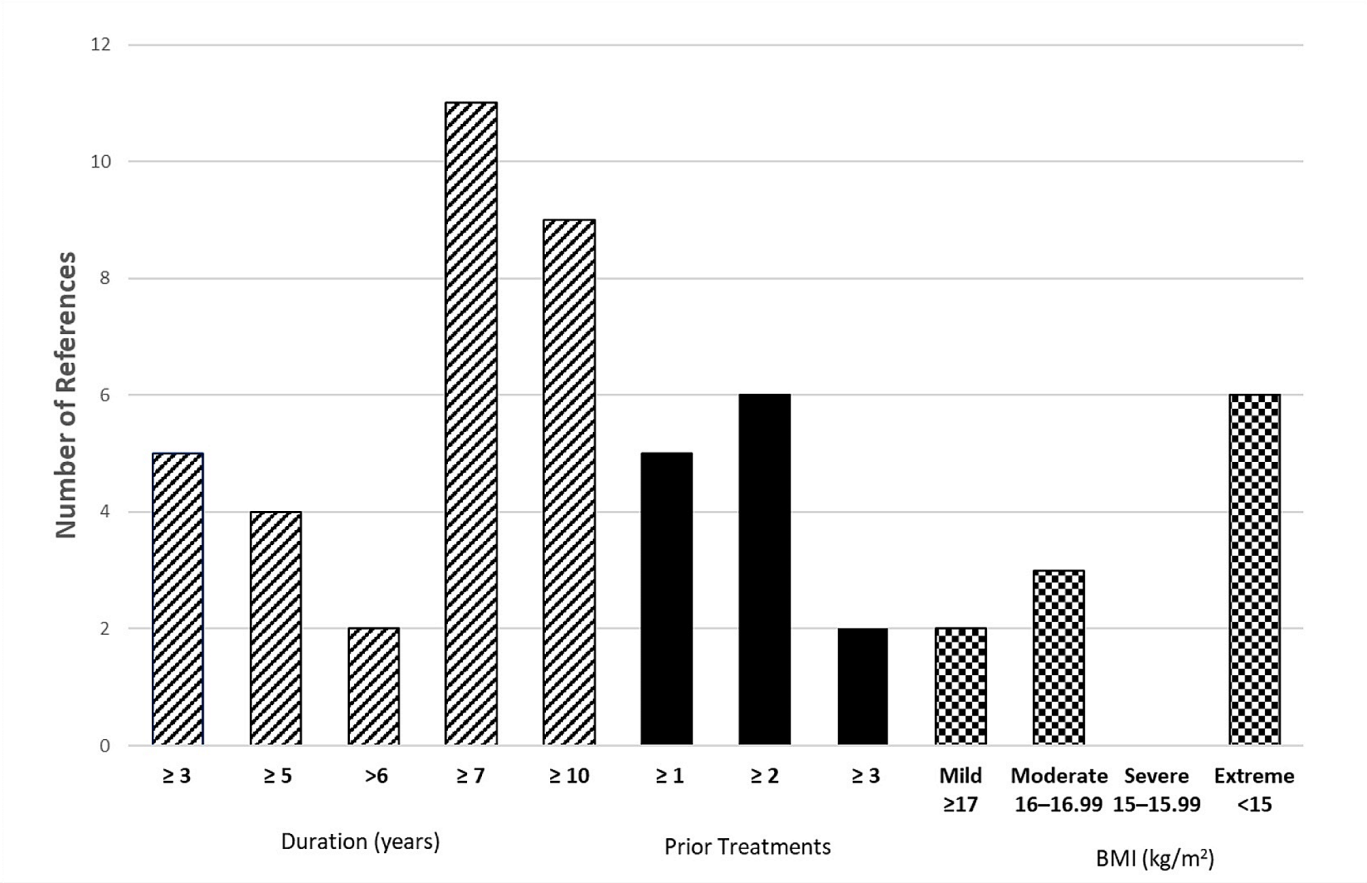
Number of references from Table 1 representing the specific duration of illness, number of previous unsuccessful treatments and body mass index (BMI) subgroups indicated either in defining severe and enduring anorexia nervosa or as inclusion criteria for participants. The totals indicated include both the references from the 2017 Broomfield review [21] and the current work

Articles were reviewed to determine which criteria are used most often in the literature in regard to the severe enduring phenotype. Specifically, articles with a central purpose of better defining a severe and or enduring/chronic AN phenotype or the need for better treatment options (for example [34, 35]), and articles including case studies or participants in one or more study groups defined as having a severe and or enduring/chronic AN phenotype (for example [36, 37]) were included. The tabulation from the Broomfield review was combined with the current total. Given that the four Dalton articles referenced the same data, they were counted as only one reference. The results are outlined in Fig. 1.

### Literature review: inclusion of participants meeting the severe and enduring AN phenotype in genetics research

**Fig. 2:**
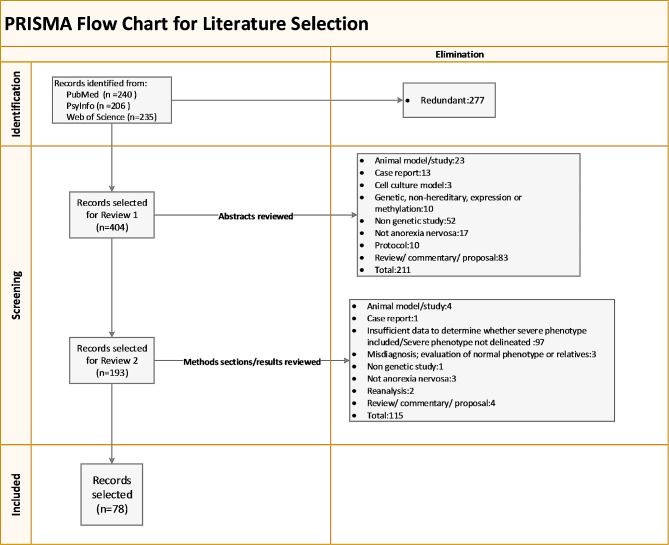
PRISMA flow diagram for the literature search

The search outlined in this section followed the process depicted in the PRISMA flow diagram [38] in Fig. 2, which captures the literature selection flow. The Preferred Reporting Items for Systematic Reviews and Meta-Analyses extension for Scoping Reviews (PRISMA-ScR) Checklist was utilized [39]. The goal was to assess whether participants meeting the criteria identified as the most widely used to define a severe and enduring phenotype are being included in genetics research, and, if included, whether these participants were assessed as an independent group.

The terms Anorexia Nervosa AND (genetic OR gene OR hereditary) in titles and abstracts were used for the following searches. Articles were limited to human subjects, and review articles were excluded. The goal was to be as inclusive as possible in the initial searches of each database. The search was limited to the last decade of published literature to assess current practices in genetics research. This span of time encompasses the five years leading up to and following the identification of the first genome wide significant locus for AN [40] and the publication of Broomfield et al., both of which were published in 2017. The inclusion dates were as follows: PubMed, 1-Jan-2012 to 6-Oct-2023 (date of search); PsycINFO, 1-Jan-2012 to 10-Oct-2023 (date of search); and Web of Science, 1-Jan-2012 to 12-Oct-2023 (date of search).

Searches of PubMed, PsycINFO and Web of Science conducted with the search criteria resulted in 240, 206 and 235 hits, respectively. Titles and keywords were reviewed, and 277 articles were eliminated for redundancy (see “identification” in Fig. 2). During the first screening, the abstracts for the remaining 404 were reviewed, and 211 were eliminated for the reasons depicted in the PRISMA diagram (“Records selected for Review 1”). The remaining 193 publications progressed to the second screening.

In the second screening, noted as “Records selected for Review 2” in the PRISMA diagram, the methods sections of the remaining 193 articles were reviewed for details on age, psychological assessments, anorexia subtype, duration of illness, prior treatment history, and other indications of disease severity. Studies did not need to specifically call out a subgroup of participants as being severe and or enduring; however, those not including participant data for at least three of the following four criteria were eliminated because they did not provide adequate information for the assessment of participant phenotype severity and intractability: (1) duration of illness; (2) body mass index (BMI); (3) prior treatment history; and (4) severity as measured by one or more clinical, social, or psychological scales. This resulted in the elimination of an additional 115 articles. A total of 78 articles were ultimately included in the information extraction process; the results are presented in Table 2.

**Table 2:** Evaluation of the inclusion of SE-AN criteria in genetic studies of anorexia nervosa. The type of AN refers to the anorexia grouping utilized by the authors, for example restricting, binge-purge, weight restored. Other EDs refer to other types of eating disorders included in the study, for example, bulimia nervosa, eating disorders not otherwise specified and binge eating disorders. Age and BMI refer to the mean age or BMI of the study participants, respectively, unless otherwise indicated. The abbreviations are detailed and annotated in the legend

Reference		Type of AN and other EDs	Age Mean (SD) years^5^	BMI kg/m^2^ Mean (SD)^5^	Duration Mean-(SD) years^5^	Prior treatment history	Gene or genetic element studied	Other measure (psychological tool, study author description)
1.	[78] Acevedo et al., 2015	AN-C, AN-WR, BN	18–67	AN-C: 17.9 ± 0.3	AN-C met all DSM-IV criteria for the previous 12 months	NA	Oxytocin	QIDS-CR; SIGH-A; YBC-EDS Female
2.	[123] Ando et al., 2012	AN-R, AN-BP, AN-P, BN, EDNOS	24.9 ± 8.39 (all ED groups)	16.0 ± 3.24^1^ 12.8 ± 2.11^2^ (all ED groups)	NA	NA	*BDNF*	TCI Female
3.	[124] Ando et al., 2014	AN-R, AN-BP, BN, EDNOS	25.1 ± 8.5	12.8 ± 2.1^2^ 16.0 ± 3.1^1^	NA	NA	*FAAH*	Female
4.	[125] Augoulea et al., 2021	AN-No Sub^3^ See “other”	15.4 ± 1.8 (typical AN) 14.4 ± 1.3 (atypical AN)	15.9 ± 1.3 (typical AN) 17.9 ± 0.8 (atypical)	NA	NA	*MTHFR*	Atypical: BMI low normal Typical: refuse to maintain a normal weight, intense fear of gaining weight, disturbed body image, amenorrhea. Female
5.	[126] Baeza-Velasco et al., 2021	AN-R, AN-BP, BN “Other ED”	All ED types 31.03 (11.02) without JHM 22.77 (2.07) with JHM	All ED types 19.31 (6.2) without JHM 20.22 (4.8) with JHM	All ED types 12.93 (9.07) without JHM 6.25 (6.73) with JHM	NA	JHM	MINI 5.0.0 EDI-2 ED without JHM: 13% male ED with JHM: no males
6.	[127] Batury et al., 2020	AN-R, AN-BP	18.0 ± 3.4 (acute) 19.7 ± 3.8 (recovered)	15.0 ± 0.3 (acute)	Not provided. Age of onset average provided.	NA “Recovered” group was noted as being previously treated.	Ghrelin and leptin receptor methylation	Defined recovered as: (1) maintain a BMI > 18.5 (if older than 18 years) or a BMI > 10th BMI percentile (if younger than 18 years, for at least three months prior to the study (2) have not binged, purged, or engaged in significant restrictive eating patterns. SIAB-EX; EDI-2 Female
7.	[128] Boehm et al., 2020	AN-R, AN-BP	16.15 ± 3.07 (All AN)	14.55 ± 1.35	Not provided. Age of onset average provided.	NA	*SLC6A4* (serotonin transporter) DNA methylation	SCL-90-R; EDI-2; BDI Female
8.	[76] Booij et al., 2015	AN-R, AN-BP, AN-P	21.5 (6.4); range: 18–38 (AN-R) 23.4 (5.6); range: 18–40 (AN-BP)	14.9 (1.8) (AN-R) 15.9 (1.1) (AN-BP)	Months 54.9 [30]; range: 12–84 (AN-R) 83.3(87.8); range: 12–336 (AN-BP)	NA	Methylation	EDEQ; CES-D Female
9.	[129] Caso et al., 2020	AN-R, AN-BP	14.77 ± 0.3 (AN-R) 15.29 ± 0.42 (AN-BP)	17.83 ± 0.46 (AN-R) 15.29 ± 0.42 (AN-BP)	Months mean 14.3	NA	Bioinflammatory response and glucocorticoid receptor expression	EDI, BITE, BSQ, HARS, MADRS, BIS, CTI Female
10.	[79] Castellini et al., 2012	AN-R, AN-BP, BN	26.54 ± 7.55 (AN- all)	16.56 ± 2.60 (AN- all)	Patient had to have reported ≥3 years of stable diagnosis per DSM-IV to be included	NA	*5-HTT* Serotonin transporter	Patients with a BMI of < 14 were considered “unsuitable for psychotherapy” and excluded from the study. EDEQ, BDI, STAI 2.9% male
11.	[130] Ceccarini et al., 2020	AN No-Sub^3^, BN, BED	22.4 ± 8.9 (AN)	14.3 ± 2.0 (AN)	NA	NA	*5-HT2AR* and *BDNF* gene variants	AN: 1.6% male BN: 4.3% male
12.	[105] Ceccarini et al., 2022	AN No-Sub^3^, noted that certain patients purged	21 (12-57) (Female) 26(9.26–18.37) (Male)	14.13 ± 2.12^2^ 21.64 ± 5.09^6^	34.9 ± 47.9 (months)	NA	*SLC25A13, PDE11A, LRP2, NNAT CD36, CACNA1C, DRD4, EPHX2, ESR1, GRIN2A, GRIN3B, LRP2, NPY4R, PTGS2, PTPN22, SGPP2*	Male and Female
13.	[131] Chang et al., 2019	AN-R, AN-BP and AN not classified	NA	NA	≥3^4^	NA	Microduplication at 15q11.2; CNV *CYFIP1; NIPA1; NIPA2*	SIAB 2.3% male
14.	[80] Chen et al., 2015	AN-No Sub^3^	19.4 ± 4.7	16.2 ± 2.7	Not provided. Age of onset average provided.	NA	5- *HTTLPR* Serotonin-transporter-linked promoter region polymorphism	EDE-Q
15.	[132] Clarke et al., 2016	AN-R AN-BP	27.4 (9.1)	17.5 (2.1) ^1^ 13.3 (1.9) ^2^	6.30 (7.63)	NA	Val66Met *BDNF* polymorphism	BSQ Female
16.	[133] Clarke et al., 2014	AN No-Sub^3^	32.4 ± 14.25	Low weight that is/was < 5th percentile for BMI^4^	Study criteria met for ≥3 years prior to entry into the study	NA	*VGLL4*	SIAB Female
17.	[81] Czerniak et al., 2013	80% AN-R mixed with OCD and anxiety	16.2 ±-2.7	14.73 ± 1.83	NA	NA	*CYP17A1*	CGI-anxiety Female
18.	[134] Dmitrzak-Weglarz et al., 2013	AN No-Sub^3^	17.5 ± 3.3	14.389 ± 1.945	Not provided. Age of onset average provided.	NA	*BDNF, NTRK2, FYN, GSK3b, GRIN2B, GRIN2A and SNAP-25*	NA
19.	[135] Dmitrzak-Weglarz et al., 2016	AN No-Sub^3^	17.5 ± 3.3	14.389 ± 1.945	Not provided. Age of onset average provided.	NA	*NR3C1 (*glucocorticoid receptor)	BDI Female
20.	[136] Dudzińska et al., 2021	AN No-Sub^3^	18.97 ± 2.54	14.9 (1.2)	NA	NA	*KAT3, KAT2, KAT1, sICAM-1, TRP*	Oral interview Female
21.	[137] Ehrlich et al., 2012	AN-R AN-BP Included acute and “weight restored”	Acute 17.88 (3.23)	Acute 14.9 (1.2)	NA	NA	Proopiomelanocortin *(POMC*) promoter-specific DNA methylation	weight-restored, subjects had to maintain a BMI ≥18.5 for > 3 months and have not binged, purged, or engaged in significant restrictive eating patterns. Female
22.	[138] Faje et al., 2012	AN No-Sub^3^	16. 7 ± 0. 22	17. 2 ± 0. 21	NA	All AN enrolled in outpatient treatment programs	*SOST*	Female
23.	[139] Fan et al., 2023	AN-R AN-BP	23.5 ± 7.0	15.6 ± 2.5	5.5 (median 4.0)	Recruited from specialized treatment centers	*Htr1b* (in mice)	EDI Female
24.	[140] Favaro et al., 2013	AN No-Sub^3^	25.0 (6.9)	15.9 [1.4]	NA	NA	Val158 Met catechol-O-methyltransferase (*COMT*)	WCST Female
25.	[141] Franzago et al., 2023	AN-R AN-BP, BN, BED, UFED, OSFED	16.0 ± 2.0 (AN-R) 18.0 ± 1.0 (AN-P)	14.4 [13.6;15.4] 15.8 [14.7;17.2]	NA	NA	DNA methylation levels at 6 CpG sites within the *SLC6A4* promoter region	EDI-3 No males included in AN groups.
26.	[142] Galimberti et al., 2013	AN-R AN-BP	24.10 ± 6.8	16.21 ± 4.02	5.95 ± 5.09	NA	Executive functioning	MINI-DIS Female
27.	[143] Gamero-Villarroel et al., 2015	AN No-Sub^3,^ BN	18.9 ± 6.2 (AN)	17.19 ± 2.01	NA	NA	*NEGR1*	EDI-2, GSI, PSDI Female
28.	[144] Gamero-Villarroel et al., 2017	AN No-Sub^3,^ BN, Obese	18.9 ± 6.2 (AN)	17.19 ± 2.01	Not provided. Age of onset average provided.	NA	*TFAP2B* and *KCTD15*	EDI-2, GSI, PSDI Female
29.	[145] Gamero-Villarroel et al., 2014	AN No-Sub^3,^ BN	18.9 ± 6.2 (AN)	17.19 ± 2.01	Not provided. Age of onset average provided.	NA	*BDNF*	EDI-2, GSI, PSDI Female
30.	[146] Gamero-Villarroel et al., 2015	AN No-Sub^3,^ BN, Obese; BED	15.50 ± 2.30 (AN)	16.40 ± 1.59 (AN)	NA	NA	Melanocortin-4 receptor (*MC4R*)	EDI-2, GSI, SCL-90-R No males included in AN group
31.	[82] Gervasini et al., 2013	AN No-Sub^3,^	26.0 ± 7.4	17.60 ± 2.33	NA	NA	dopamine receptors (*DRD2, DRD3, and DRD4*), transporters (*DAT1*) and metabolizing enzymes (*COMT*) gene polymorphisms	EDI-2, SCL-90R, GSI Female
32.	[147] Gervasini et al., 2018	AN No-Sub^3,^ BN	18.9 ± 6.2 (AN)	17.54 ± 2.27 (AN)	NA	NA	Dopamine D4 Receptor (*DRD4*)	PSDI, GSI, PST, SCL-90R Caucasian female
33.	[148] Gervasini et al., 2018	AN No-Sub^3^, BN, BED	16.94 ± 4.58	17.54 ± 2.27	NA	NA	Val158Met catechol-O-methyltransferase (*COMT*)	GSI (SCL-90R) PST (SCL-90R) and PSDI (SCL-90R) Female
34.	[83] González et al., 2021	AN No-Sub^3^	16.96 ± 4.11	17.33 ± 2.11	NA	NA	Cannabinoid Receptor (*CNR1, CNR2)*	EDI-2, SCL-90R, PST, PSDI Female
35.	[84] González et al., 2021	AN-R, AN-BP, BED	16.98 ± 4.21 (AN)	17.32 ± 2.07 (AN)	NA	NA	Fat mass obesity (*FTO*)	EDI-2, GSI, PST, PSDI Caucasian, female
36.	[149] He et al., 2023	AN-R, AN-BP	18.96 ± 4.14	15.22 ± 2.08	29.73 ± 30.99 (months)	NA	*SLC6A4* Hypermethylation	EDE-Q, BDI, BAI Chinese Han descent, female
37.	[75] Hernández et al., 2016	AN-R and EDNOS-AN AN-BP, BN-P, BN-NP, and EDNOS-BN	18.23 ± 4.4 (for all EDs)	NA	4.4 (4.14) (not divided out-all EDs)	NA	Serotonin receptor 1Db (*HTR1B*)	HAM-A, HAM-D, SCID-1 7.9% Male Female
38.	[150] Hernández-Muñoz et al., 2020	AN-R, BN	18.08 (3.89) (AN-R)	15.66 (1.51) (AN-R)	4.03 years (4.44)	NA	Serotonin transporter (*SLC6A4*)	EDI-2, YBC-EDS Female
39.	[151] İnan-Erdoğan et al., 2019	AN No-Sub^3^	14.50 ± 1.49	15.82 ± 2.44	NA	NA	Vitamin D receptor (VDR) (VDRBsml, VDRFokl) and estrogen receptor (ESR) (ESR1Xbal, ESR1Pvull)	20% male
40.	[101] Iranzo-Tatay et al., 2022	Monozygotic twin pairs, discordant for AN And AN-R AN-BP group of nontwins	20.42 (7.39) (MZ) 20.14 (7.08) (Nontwins)	16.75 (1.43) (MZ twins) 15.62 (0.95) (Nontwins)	NA	NA	Genome-wide methylation	Female
41.	[50] Johansson et al., 2022	AN-R, AN-BP, AN-EDNOS, BED, BN	19.44 (6.68) (AN-R) 21.79 (7.00) (AN BP)	15.91 (1.39) (AN-R) 16.52 (1.49) (AN-BP)	≥ 5 (follow-up time)	All patients registered in the study had undergone at least one treatment attempt	Polygenic scores (PGSs)	Defined severity as CIA ≥ 18 Created an index of Severe Enduring = high CIA score and follow-up time ≥ 5 years 1.6% male
42.	[85] Kang et al., 2017	AN No-Sub^3^	19.05 ± 4.52	Three genetic subgroups 15.97 ± 2.40 16.65 ± 2.43 15.92 ± 2.82	Not provided. Age of onset average provided.	NA	5-HT_2A_ receptor gene promoter polymorphism‐1438G/A	EDE-Q 6.0 Female
43.	[152] Kesselmeier et al., 2018	AN-R AN-BP. Also looked at lean controls without AN (BMI < 15)	15 [14, 17] (Q1 Q3) (AN-R) 12.7 (12.1, 14.0) ) (Q1 Q3) (AN-BP)	13.7 (13.0, 14.5) (Q1 Q3) (AN-R) 12.7 (12.1, 14.0) (Q1 Q3) (AN-BP)	NA	NA	*TNXB* hypermethylation; high-throughput DNA methylation derived from whole blood	Female
44.	[153] Kim et al., 2013	AN (5 AN-R, 1 AN-BP)	34 22 39 19 24 22 Age of each patient	(13.8, 18.9) (14.6, 18.2) (16.2, 18.1) (14.8, 17.6) (14.9, 18.4) (17.1, 19.3) Before and after treatment for each patient	NA	NA	Gene expression before and after weight restoration	Detailed blood chemistries provided. Female
45.	[86] Kim et al., 2014	AN No-Sub^3^	24.73(10.73)	15.06 (2.58)	NA	NA	Methylation status of the oxytocin receptor (*OXTR*)	EDE-Q, AQ Female
46.	[154] Kim et al., 2015	AN-R, AN-BP, BN	21.70 (7.06)	14.81 (2.18)^1^ 13.80 (1.87)^2^ 20.97(3.89)^6^	3.27 (4.88)	NA	*OXTR* rs5357	EDE-Q, BDI, STAI-State and Trait, BIS/BAT Korean females
47.	[155] Kucharska et al., 2019	AN-R AN-BP	22.38(2.76)	15.02(1.33)	Not provided. Age of onset average provided.	NA	Oxytocinergic system gene polymorphisms	BDI-II, EAT-26, TAS-20 Female
48.	[74] Kushima et al., 2022	AN-R, AN-BP, ARFID	29.2 ± 9.4 15–23 for those with NDD-CNVs	Lifetime minimum BMI < 15 kg/m^2, 4^ 10.6 to 14.6 for those with NDD-CNVs	Not provided. Age of onset provided.	Noted that they did record the previous number of hospitalizations but did not provide this in the article or supplementary data	Neurodevelopmental disorder CNVs	All patients required hospitalization, were specifically noted as having “severe ED” which was defined by a lifetime minimum BMI < 15 kg/m^2^ Female
49.	[156] Lawson et al., 2012	AN-R (if reported more than one B and P per month in the 3 months were excluded)	21.7 ± 0.7 (AN) 23.2 ± 0.8 (AN-Weight restored)	17.7 ± 0.3 BMI (AN) 22.1 ± 0.7 BMI (AN-WR)	52.7 ± 11.2 months (AN) 36.3 ± 6.6 months (ANWR)	NA	Oxytocin secretion and Insular Cortex Hypoactivation	EDE-Q, BDI, STAI Female
50.	[157] Lin et al., 2020	AN-R	25 (19, 42)-Nonremission 32 (21, 41) Remission (min-max)	≤18^4^ 16.5 (12, 16.8) Nonremission 20.5 (19, 25) Remission (min-max)	≥2^4^ 7.7 (1, 26) Nonremitted 13 (6, 240 (Remitted) min-max	NA	*CADM1* rare variants	EAT-26, EDI-2 Female
51.	[158] Lutter et al., 2017	AN-R, AN-BP, BED	18–60 with a median age of 24 (for all ED)	15.71 (2.2) AN-R 16.84 (0.8) AN-BP	NA	NA	Novel damaging genetic variants	4% Male Female
52.	[159] Müller et al., 2012	AN No-Sub^3^, BN	25.02 ± 6.26 (Italian) AN 24.03 ± 6.19 (Spanish) AN 18.35 ± 6.51 (German 1 AN) 16.20 ± 2.00 (German 2 AN, first admission) 33.03 ± 7.47 (German 2 AN, current)	14.77 ± 2.11 (Italian) AN 16.97 ± 1.91 (Spanish) AN 16.07 ± 2.92 (German 1 AN) 14.50 ± 1.60 (German 2 AN, first admission) 20.83 ± 2.40 (German 2 AN, after recovery)	NA	NA	Fat Mass and Obesity-Associated Gene (*FTO*) rs9939609	2.8% Male Female
53.	[87] Neyazi et al., 2019	AN-R, AN-BP	30.26 ± 9.31 (full AN R or BP) 26.84 ± 6.84 (partial AN R or BP) 24.92 ± 5.97 (full recovery)	16.26 ± 0.94 (full AN R or BP) Time 0 17.08 ± 0.85 (partial AN R or BP) Time 0 17.03 ± 0.82 (full recovery) Time 0	Although the original study is based on [160] divided based on durations of > 6 years and ≤6 years, this article did not provide these details.	The article did not note prior treatment, however as it included a follow-up at 12 months post treatment completion, one could consider those with full AN at that time point to have had one unsuccessful treatment.	Leptin gene (*LEP*) and the leptin receptor gene (*LEPR*) DNA promoter methylation	Severity of AN and outcome were measured by the PSR based on the patient’s SIAB-EX interview. PSR scores range from 1 (no symptoms of AN) to 6 (severe symptoms of AN that require admission) Full recovery = PSR score of 1 or 2 and BMI > 18.5 Full AN = PSR score of 5 or 6 and BMI of ≤17.5; and Partial AN included all other cases. Female
54.	[161] Ortega et al., 2016	AN-R, AN-BP	24.0 (5.3)	17.4 (1.4)	5.5 (SD = 5.3)	NA	*TAS2R38*	EDI-2, SCL-90R Female
55.	[162] Phillipou et al., 2022	AN-C (current) AN-wr (weight recovered)	22.55 (3.10) (AN-c) 22.45 (2.79) (AN-wr)	16.72 (1.48) AN-c	5.74 (3.94) AN-c 4.34 (2.98) AN-wr	NA	Anxiety, heritability, perfectionism	MINI, EDE-Q, DASS, BSAT, MPS Female
56.	[88] Plana et al., 2019	AN-R and AN-P but not delineated in analysis for genetics	14.4 (2.2) current for those with very early onset AN 16.3 (1.4) current for those with early onset AN	Mean lowest BMI in the acute phase of AN was 16.1 (1.5)	NA	NA	Genetic pleiotropy related to the serotonergic system	LOI-CV, CDI, CAPS, EAT-40 Very-early onset AN = onset < 13 years Early 13–18 years Very early: 38% male Early: 23% male
57.	[163] Rudolph et al., 2023	AN No-Sub^3^	26.3 ± 9.6 Group 1 27.8 ± 9.4 Group 2	12.7 ± 1.8 Group 1 14.5 ± 1.8 Group 2	NA	NA	Neuronatin expression	GAD-7, PSQ-20, PHQ-9, EDI-2 Female
58.	[164] Sala et al., 2018	AN No-Sub^3^	26.96 (6.92) (GG genotype) 27.15 (9.04) (GA/AA genotype)	19.36 (2.80) (GG genotype) 20.34 (3.28) (GA/AA genotype)	NA	NA	*OXTR*	QIDS-CR, SIGH-A, YBC-EDS, EAT-26 EAT-D EAT-B EAT-O BSQ Female
59.	[165] Schroeder et al., 2012	AN-R, AN-BP, BN	25.25 (13.2) (AN) wrist cutting (WC) 26.69 (10.3) (AN) non-wrist cutting (NWC)	17.35 (1.2) (AN WC) 15.50 (2.0) (AN NWC)	11.75 (11.5) (ANWC) 9.20 (11.0) (ANNWC)	NA	The cannabinoid 1 (CB 1) receptor is the primary mediator of the endocannabinoid (EC) system	EDI-2, BDI Female
60.	[89] Scott-Van Zeeland et al., 2014	AN-R AN-BP	26.9 (8.0) Discovery 28.0 (8.5) Pooled replication	18.8 (2.8) Discovery 18.3 (2.7) Pooled replication	See Other Measure column 9.9 (6.9) Discovery 10.3 (7.8) Pooled replication	NA	Epoxide Hydrolase 2 (*EPHX2*)	BDI, YBC-SDS, STAI EPHX2 Male and female European descent
61.	[166] Shih et al., 2016	AN-R (ruled out BP and Binge) AN-I = AN-“ill” AN-Rec = AN-“recovered”	22.2 ± 4.8 (AN-I) 24.2 ± 5.7 (AN-Rec)	≤17.5 (AN-I) ≥ 18 (AN-Rec)^4^ 14.3 ± 1.4 (AN-I)^1^ 21.0 ± 1.763 (AN-Rec)^1^ 12.3 ± 1.6 (AN-I)^2^ 14 ± 1.8 (AN-Rec)^2^	Study diagnostic criteria were met for at least 3 years before study entry	NA	Epoxide Hydrolase 2 (*EPHX2*) and Lipidomic Profiles	AN- “recovered” defined as having an absence of ED symptoms in the past year including maintenance of a BMI of 18 or greater. BDI, STAI, TCI Also measured Total cholesterol and HDL European descent; Female
62.	[167] Shimizu et al., 2020	AN-R AN-BP	28.42 ± 12.39 AN-R 32.43 ± 8.19 AN-BP	12.81 ± 2.00 AN-R 12.17 ± 1.58 AN-BP	2114.08 ± 3255.41 (days) AN-R 2629.29 ± 2037.48 (days) AN-BP	NA	Fatty acid metabolism	Female
63.	[77] Steiger et al., 2019	AN-R AN-BP	24.88 ± 8.39 (18–53 min-max) (AN-R active) 23.41 ± 5.28 (18–43 min-max) (AN-BP active)	14.39 ± 1.72 (10.80–17.54 min-max) (AN-R active) 15.78 ± 1.20 (13.09–17.81 min-max) (AN-BP active)	96.00 ± 98.91 (12–456 (min-max)) (AN-R active) Months 78.44 ± 57.68 (12–192 (min-max)) (AN-BP active) Months	Did not note whether those in the “remitted” group received treatment or whether those in the active groups received previous treatment	Epigenome-wide study of DNA Methylation	Remitted: Previous AN per DSM; at time of study did not meet criteria for AN or BN and had maintained a self-reported BMI of ≥18 for at least 12 months. EDE, EDE-Q Female
64.	[168] Stergioti et al., 2013	AN No-Sub^3^	15.8 ± 1.6	16.25 ± 1.41	NA	NA	*ESR1, CTR*	Female
65.	[169] Subramanian et al., 2018	AN-R AN-binge eating. Individuals with binge eating/purging-AN were excluded	24 (6.1) AN-R	16 (1.9) AN-R	NA	NA	Histone deacetylase 4 (*HDAC4*) methylation	Female
66.	[170] Svedlund et al., 2022	AN No-Sub^3^	16–24	15.2 (1.3) group 2 15.6 (0.9) group 2 15.6 (0.5) group 2 At start of treatment	27 months (3–120 min-max, months)	NA	Fat Mass and Obesity-Associated Gene (*FTO*)	Female
67.	[171] Tenconi et al., 2016	AN No-Sub^3^	22.64 ± 6.9	17.58 ± 3.0^1^ 14.96 ± 2.1^2^ 21.21 ± 2.8^6^	NA	NA	*5-HTTLPR*	H-SCL-90, STAI Female
68.	[172] Thaler et al., 2020	AN-R, AN-BP Active-Act Remitted- Rem	23.53 (5.78) AN-Act 27.43 (5.54) AN-Rem	15.21 (1.65) (AN-Act) 21.52 (2.05) (AN-Rem)	82.54 (64.56) range 12–300 months (AN-Act) 86.0 (41.21) range 24–216 months (AN-Rem)	Did not note whether those in the “remitted” group received treatment or whether those in the active groups received previous treatment	Oxytocin receptor (*OXTR*) methylation	AN-Rem = BMI ≥18.0, and they reported no restriction, binge eating, or purging for the past 12 months. EDE-Q Female
69.	[173] Tremolizzo et al., 2014	AN-R (not clear whether this was strictly defined)	15.5 ±1.4	15.5 ± 2.1	NA	NA	Whole-blood global DNA methylation and serum hormones	EDI-3, STAI-Y, CDI Female
70.	[90] Yilmaz et al., 2014	AN-R, AN-BP, BN	26.1 ± 8.5 (AN)	Minimum illness-related lifetime BMI < 18.5^4^ 18.05 ± 2.71^1^ 13.82 ± 1.95^2^	≥3 y^4^	NA	Leptin, melanocortin, and neurotrophin genes	NA Female
71.	[174] Yilmaz et al., 2023	AN No-Sub^3^	Evaluation at ages 14 and 16	NA	NA	NA	Transdiagnostic polygenic scores	YRBSS Male and female
72.	[175] Zhang et al., 2013	AN No-Sub^3^	19.3 ± 4.9	NA	NA	NA	Estrogen receptor 1 gene (*ESR1*) rs2295193 polymorphism	NA 5.6% male
73.	[176] Zhang et al., 2022	AN No-Sub^3^	Age at first ED diagnosis without ASD: 18.87 (4.36) with ASD: 18.45 (4.52)	Minimum BMI during AN: Without ASD: 15.59 (1.87) With ASD: 14.76 (2.34)	NA	Inpatient treatment 698/3055 (23%) without ASD 62/134 (46%) with ASD. Number of inpatient days without ASD 28.36 (110.12) with ASD 121.90 (393.36)	Autism Spectrum Disorder (ASD)	EDQ, GAF, CIA, CGI 1.9% male without ASD 5.2% male with ASD
74.	[177] Zhang et al., 2023	AN No-Sub^3^ BN, BED, EDNOS	Age at 1st diagnosis: 17.97 ± 4.22	Lifetime minimum BMI 17.41 ± 2.71	NA	NA	Association with schizophrenia	EDE-Q 5.8% male
75.	[178] Zheng et al., 2022	AN No-Sub^3^	19.52 ± 9.08 (AN-acute) 33.09 ± 9.52 (AN-recovered)	15.71 ± 1.81 (AN-acute) 19.91 ± 2.41 (AN-recovered)	NA	NA	Polypyrimidine Tract Binding Protein 2 gene (*PTBP2*).	All female in AN groups
76.	[179] Zheng et al., 2023	AN No-Sub^3^	19.47 (7.68) (AN-acute) 27.09 (9.88) (AN-recovered)	15.6 (1.8) (AN-acute) 20.56 (2.75) (AN-recovered)	NA	NA	Lipocalin-2 (*LCN2*); Melanocortin 4 receptor (*MC4R*)	Female
77.	[180] Ziora-Jakutowicz et al., 2021a	AN No-Sub^3^	15.06 ± 1.57	15.19 ± 1.67	NA	NA	*ADIPOQ c.45 T >* G and *ADIPOQ c.276* G > T polymorphisms in the adiponectin coding gene	Female
78.	[181] Ziora-Jakutowicz et al., 2021b	AN No-Sub^3^	15.06 ± 1.57	15.19 ± 1.67	NA	NA	*RETN c.62*G > A and *RETN c.-180*C > G polymorphisms in the resistin coding gene	Female

**AN-R-**Anorexia Nervosa restricting type; **AN-BP**-Anorexia Nervosa binge purge type; **AN-C-**Currently have Anorexia Nervosa; **AN-WR**- Weight Restored anorexics; **ASD**: Autism Spectrum Disorder; **AQ**- Autism Spectrum Quotient; **BAI**-Beck Anxiety Inventory; **BED-**Binge Eating Disorder; **BDI** = Beck Depression Inventory; **BDNF**- Brain-Derived Neurotrophic Factor; **BSAT**- Brixton Spatial Anticipation Test; **BSQ**- Body Shape Questionnaire; **BIS**- Barratt Impulsiveness Scale; **BITE-** Bulimic Investigatory Test, Edinburgh; **BMI**-Body Mass Index; **BN**-Bulimia Nervosa; **CAPS**-Child and Adolescent Perfectionism Scale; **CDI** Children’s Depression Inventory; **CES-D**- Center for Epidemiological Studies-Depression scale; **CGI**- Clinical Global Impression Anxiety scale; **CIA**-Clinical Impairment Assessment; **CNV**-Copy Number Variant; **CTI**-Childhood Trauma Interview; **DASS**-Depression Anxiety Stress Scale; **EAT**- Eating Attitudes Test- **EDEQ**-Eating Disorder Examination-Questionnaire; **EDI**- Eating Disorders Inventory; **EDNOS**-Eating Disorder Not Otherwise Specified; **GAD-7-**General Anxiety Disorder-7; **GAF-**Global Assessment of Functioning; **GSI-**Global Severity Index; **HAM-A HAM-D**- Hamilton Scales for Anxiety and Depression; **HARS**- Hamilton Anxiety Rating Scale; **H-SCL-90**- Hopkins Symptoms Checklist; **JHM-** Joint hypermobility; **LOI-CV**-Leyton Obsessional Inventory-Child Version **MADRS**- Montgomery-Asberg Depression Rating Scale; **MINI**- Mini International Neuropsychiatric Interview; **MPS**- Multidimensional Perfectionism Scale; **MTHFR**-methylenetetrahydrofolate Reductase; **MZ**-Monozygotic; **NA**- Not applicable (and/or provided); **NDD**- Neurodevelopmental Disorder; **OCD**- Obsessive Compulsive Disorder; **PHQ-**9- Patient Health Questionnaire-9; **PSDI**- Positive Symptom Distress Index; **PSQ-20**- Perceived Stress Questionnaire; **PST**- Positive System Total; **QIDS-CR**-Quick Inventory of Depressive Symptomalogy; **SCID**- Structured Clinical Interview for Mental Disorders; **SCL-90-R**- Symptom Checklist 90 Revised; **SIAB-EX**- Structured Interview for Anorexia and Bulimia Nervosa; **SIGH-A**-Structured Interview Guide for the Hamilton Anxiety Scale; **STAI-Y**- State-Trait Anxiety Inventory form; **STI-TCI**-Temperament and Character Inventory; **TAS**- Toronto Alexithymia Scale; **TCI-**Temperament and Character Inventory; **WCST**-Wisconsin Card Sorting Task; **YBC-EDS**-Young-Brown Obsessive-Compulsive Symptoms; **YRBSS**- Youth Risk Behavior Surveillance System

(1) Current. (2) Lifetime minimum (3) Anorexia with no subtyping (restricting vs. binge purge) (4) Inclusion criteria (5) Unless otherwise indicated (6) Lifetime maximum

The data were extracted by reviewing both the methods and results sections of each paper for the following participant data: (1) mean duration of illness in years; (2) mean BMI in kg/m^2^; (3) prior treatment history; (4) and severity as measured by one or more clinical, social, or psychological scales. Participant gender, mean age, and groups of eating disorders included in the studies (i.e., AN-restricting, AN-binge purge, bulimia, binge eating) were also extracted. A second reviewer, using the RANBETWEEN function in Microsoft Excel, selected 10% of the articles at random from Table 2 to review for meeting inclusion criteria and accuracy of the data extracted.

## Results

### Defining severe enduring anorexia nervosa in the research literature

A review of the literature revealed that the terms severe, chronic, and enduring identified by Broomfield et al., in 2017 [21] continue to be widely used to label the more intractable AN phenotype. How these labels are defined in the literature, when they are defined, continues to vary greatly. The age of study participants, BMI, duration of illness, and previous treatment history were extracted from each reference and are recorded in Table 1.

The primary inclusion criteria presented in the articles reviewed were as follows:

1. Duration:

The Broomfield review [21] identified duration as the primary criterion used to define the severe and enduring AN phenotype, and this continues to be true. Several articles reviewed included duration of illness as a criterion for inclusion in their study or clearly delineated a subgroup using duration as one criterion. The stringency of how duration was measured varied.

In their audit of care received by patients with “early stage” versus “severe and enduring” AN, Ambwani et al. [36] defined a duration of < 3 years for early stage and ≥7 years for severe and enduring AN, as recommended by Robinson et al. and Touyz et al. [41, 42]. This was also the case for Calugi et al. [43], who used ≥7 years in their study of cognitive behavioral therapy effectiveness. The patient described in the case study by Voderholzer et al. [44] had AN for seven years. In the four papers by Dalton et al. studying the impact of transcranial magnetic stimulation on severe and enduring AN, the duration inclusion criterion for study participation was ≥3 years of AN symptoms [45–48]. Whereas Knyahnytska et al. [49] included a duration of > 5 years as a criterion for treatment resistance in their insula H-coil transcranial stimulation therapy study. In the selection of a subset of participants from the Anorexia Nervosa Genetics Initiative (ANGI) to include in their assessment of the polygenic association of severity and long-term outcome in AN, Johansson et al. [50] included in their criteria for the severe enduring subtype a ≥ 5 year follow-up time, defined by the authors as years between initial registration and ANGI recruitment. Finally, in two of the three studies evaluating the effectiveness of deep brain stimulation, an illness duration of ≥ 10 years was required for participant inclusion [51, 52], with the third requiring > 7 years [53]. Case study, clinical trial and study participants included in groups indicated as manifesting a severe and enduring phenotype tended to have illness of longer duration. For example, participants in the Calugi et al. [43] study had a mean duration of 12.3(4.7 SD) years, and the three case study subjects had illness durations of 7 [44], 11 [54], 25 [55], and 26 [37] years.

Position papers, commentaries, and reviews also varied greatly in defining duration requirements. For example, in their German language case study on palliative care for severe AN, Westermair et al. [56] proposed a long duration of illness, e.g., 10 years, as a criterion, whereas Hay and Touyz [22] and Herpetz-Dahlmann [57] used a duration of > 3 years. Other authors fell between the two extremes; Bianchi et al. [58] defined severe and enduring AN participants as those who had the disorder for six years or more, and Marzola et al. [59] used a seven-year demarcation. However, these two papers also proposed that duration should not be used alone when defining AN severity. The usefulness of duration as a criterion was also questioned by Wildes et al. [60]. In an attempt to define the severe and enduring phenotype empirically, Wildes found no evidence for a chronic subgroup of AN, instead proposing that this group may be better classified on the basis of impact on quality of life and severity of injurious behaviors. As indicated in Fig. 1, a duration of 7 or more years was used most frequently, followed by 10 years.

2. Severity:

Body mass index (BMI):

The DSM-5 defines four levels of AN severity: mild, BMI greater than 17 kg/m^2^; moderate, BMI of 16–16.99 kg/m^2^; severe, BMI of 15–15.99 kg/m^2^; and extreme, BMI of less than 15 kg/m^2^ [61]. Once again, the literature indicates a wide range of BMIs in articles attempting to define severe and enduring AN and/or for participation in studies targeting this group of individuals. The two studies of deep brain stimulation with duration criteria of ≥ 10 years for participation also had BMI requirements falling into the DSM extreme category [51, 52]. Deep brain stimulation involves a high degree of risk, and the authors delineated that only individuals with the most severe cases should be included. Similar to duration of illness, participants included in groups indicated as manifesting a severe and enduring phenotype in case studies, clinical trials and studies, tended to have substantially lower BMIs than required per the inclusion criteria. For example, participants in the Bemer et al. bone mineral density (BMD) study had a mean BMI of 12.60 ± 1.60 kg/m^2^, which was well below the < 16 kg/m^2^ criteria [62].

Notably, several studies included a low weight cutoff for participation. For example, in their transcranial magnetic stimulation studies, Dalton et al. [45–48] required a BMI > 14 kg/m^2^ for participation. The reason provided in the study protocol for the low weight cutoff was “safety precaution” [63]. The deep brain stimulation studies conducted by Park et al. [64] required that participants be severely underweight but with a low-weight BMI criterion of > 13 kg/m^2^. Although reasons were not given for the low weight cutoff, they stated that participants needed to have a BMI > 13 kg/m^2^ for surgery, which is understandable given its invasive nature.

Again, as with duration of illness, the literature suggests that BMI should not be used as the sole determinant of severity in AN. In their editorial on the challenges of defining severe and enduring AN, Hay and Touyz [22] recognized the utility of the DSM-5 BMI severity categories but also noted that for those with unremitting AN for a decade or more, having a BMI above the DSM severe range is still associated with marked morbidity.

Psychological assessment:

All the studies reviewed included an assessment of symptoms such as psychological stress, disordered eating, depression, anxiety, obsessiveness, and quality of life. For example, Wildes et al. [60], used the Research and Development Corporation (RAND) 36-Item Health Survey 1.0 (SF-36) to measure health-related quality of life, and found that these scores better classified AN subgroups than BMI and duration of illness. A score of ≤45 on the Global Assessment of Functioning (GAF) found in the DSM-4, which assesses the severity of mental illness [65], was used by Oudijn et al. [51] for inclusion in their deep brain stimulation studies. A plethora of tools was used in assessing eating disorder pathology, with the Eating Disorder Examination Questionnaire (EDE-Q) [66] and/or various iterations of the EDE-Q being the most prevalent.

3. Treatment response:

Lack of positive response to prior treatment, variously described as treatment resistance, treatment refractoriness, and failure to respond, was also included in assessing AN severity in several of the articles. The number and type of previous treatments required for inclusion in studies varied. For inclusion in deep brain stimulation studies, Park et al. [67] required a lack of positive response to ≥2 “typical modes” of treatment, as did Oudijn et al. [51]. The participant inclusion criteria used by Dalton et al. [48] for transcranial stimulation studies included the need to have completed at least one “previous course of National Institute for Health and Care Excellence” recommended “specialist psychotherapy or specialist day-patient or inpatient treatment”. The clearest classification criterion for treatment resistance was proposed by Hay and Touyz et al. [68]: “exposure to at least two evidence-based treatments delivered by an appropriate clinician or treatment facility together with a diagnostic assessment and formulation that incorporates an assessment of the person’s eating disorder health literacy with an assessment of the person’s stage of change”, which was referenced in the reviews of treatment options for those with severe enduring AN by Zhu et al. and Wonderlich et al. [20, 69]. In contrast, Smith and Woodside [70] defined treatment resistance as “patients with two or more incomplete inpatient admissions and no complete admissions”. Emphasis was placed on patients failing to complete treatment rather than the treatment failing to help patients, although the authors did note that approximately 10% of patients treated at their inpatient facility were “unable to benefit”. As indicated in Fig. 1, the criterion of two or more treatment attempts was most frequently used.

In summary, the literature indicates that a combination of assessments and criteria, including an illness duration of ≥ 7 years, lack of positive response to at least two previous evidence-based treatments, a BMI meeting the DSM-5 for extreme AN, and an assessment of psychological and/or behavioral severity indicating a significant impact on quality of life, were the most prevalent means of defining the severe and enduring AN phenotype. As the DSM-5 includes clear definitions of severe and extreme BMI (15–15.99 kg/m^2^ and < 15 kg/m^2^, respectively), the criteria for severe BMI were also used in assessing the genetics literature in the following section.

### Inclusion of participants meeting severe enduring anorexia nervosa-defining criteria in studies of anorexia nervosa genetics

The 78 articles identified as meeting the search criteria defined in the methods section were assessed for whether the following inclusion criteria were used and how they were defined:

1. Duration of illness,
2. Prior treatment history,
3. BMI, and.
4. Severity as measured by one or more clinical, social, or psychological scales.

As mentioned previously, neither the statistical strength of the studies nor the study outcomes were assessed, as the purpose was to determine whether genetic studies included those meeting the severe and enduring phenotype criteria defined in the first aim through assessing prevalence of use in the literature. The studies consisted of Genome-Wide Association Studies (GWAS) as well as analyses of polymorphisms, expression, and gene methylation, including but not limited to the *leptin* (*LEP*) and the *leptin receptor* (*LEPR*) genes, the *fat mass and obesity-associated* gene (*FTO*), and the *oxytocin receptor* (*OXTR*) gene [16, 71–73]. The gender of the study participants was also recorded where reported (Table 2).

Most of the 78 articles, including those specifically stating that the study was of severe AN, did not include criteria defined in the first aim. Most notably, only one article specifically stated that participants included had at least one prior treatment attempt [50].

Of the 71 studies reporting mean BMI, the mean BMI for all groups was 15.73 kg/m^2^ (SD 1.48). For 15 studies (21%), the mean BMI was > 17 kg/m^2^ (mild DSM-5). Sixteen studies (22%) had a mean BMI of 16–16.99 kg/m^2^ (moderate DSM-5). Twenty-three studies (32%) had a mean BMI of ≤15.99 kg/m^2^ (severe DSM-5), and 17 studies (21.8%) included at least one group with a mean BMI of ≤15 kg/m^2^, required to meet the DSM-5 definition of extreme AN. Only one study included a lifetime minimum BMI of ≤15 kg/m^2^ as an inclusion criterion [74].

The duration of illness and or minimum duration required for inclusion in studies were reported for 23 (29%) of the 78 articles. Of those 23 studies, 3 (13%) had participants with a mean duration of illness ≤ 3 years, 12 (52%) had a mean of 3.1–6.99 years, and 6 (26%) had a mean of ≥ 7 years. Five of the 23 studies required a duration of illness ≥3 years as a participant inclusion criterion. None of the articles identified required duration of illness ≥7 years as an inclusion criterion.

Assessment of psychological stress, disordered eating, depression, anxiety, obsessiveness, and quality of life was another facet of defining the severity of AN in the studies evaluated. Across the 54 studies identifying defined assessment modalities, 38 different tools, checklists and guidelines were used in various combinations, including the following: Hamilton Anxiety Rating Scale (HARS), Clinical Global Impression anxiety scale (CGI), State-Trait Anxiety Inventory form (STAI); depression: Beck Depression Inventory (BDI), Children’s Depression Inventory (CDI), Montgomery-Asberg Depression Rating Scale (MADRS); alexithymia: Toronto Alexithymia Score (TAS); obsessive-compulsive and impulsive symptoms: Young-Brown Obsessive-Compulsive Symptoms (YBC-EDS), Leyton Obsessional Inventory-Child Version (LOI-CV); Barratt Impulsiveness Scale (BIS); and perfectionism: Child and Adolescent Perfectionism Scale (CAPS). Numerous eating disorder assessment tools, including the Eating Disorders Inventory (EDI), Eating Disorder Examination Questionnaire (EDE-Q), Eating Attitudes Test (EAT), and the Structured Interview for Anorexia and Bulimia Nervosa (SIAB) were also used. Table 3 shows a list of tools and how often they were used.

**Table 3:** List of tools ranked by use

Assessment Tool		# of Papers
EDI	Eating Disorders Inventory	19
EDEQ	Eating Disorder Examination-Questionnaire	12
BDI	Beck Depression Inventory	9
GSI	Global Severity Index	8
PSDI	Positive Symptom Distress Index	7
STAI	State-Trait Anxiety Inventory form	7
SCL-90	Symptom Checklist 90 Revised	6
EAT	Eating Attitudes Test	4
PST	Positive System Total	4
SIAB-EX	Structured Interview for Anorexia and Bulimia Nervosa	4
YBC-EDS	Young-Brown Obsessive-Compulsive Symptoms	4
BSQ	Body Shape Questionnaire	3
MINI	Mini International Neuropsychiatric Interview	3
BIS	Barratt Impulsiveness Scale	2
CDI	Children’s Depression Inventory	2
CGI	Clinical Global Impression Anxiety scale	2
CIA	Clinical Impairment Assessment	2
QIDS-CR	Quick Inventory of Depressive Symptomalogy	2
SIGH-A	Structured Interview Guide for the Hamilton Anxiety Scale	2
TCI	Temperament and Character Inventory	2
AQ	Autism Spectrum Quotient	1
BAI	Beck Anxiety Inventory	1
BITE	Bulimic Investigatory Test, Edinburgh	1
BSAT	Brixton Spatial Anticipation Test	1
CAPS	Child and Adolescent Perfectionism Scale	1
CES-D	Center for Epidemiological Studies-Depression scale	1
CTI	Childhood Trauma Interview	1
DASS	Depression Anxiety Stress Scale	1
GAD-7	General Anxiety Disorder-7	1
GAF	Global Assessment of Functioning	1
HAM-A, HAM-D	Hamilton Scales for Anxiety and Depression	1
HARS	Hamilton Anxiety Rating Scale	1
H-SCL-90	Hopkins Symptoms Checklist	1
LOI-CV	Leyton Obsessional Inventory-Child Version	1
MADRS	Montgomery-Asberg Depression Rating Scale	1
MPS	Multidimensional Perfectionism Scale	1
PHQ-9	Patient Health Questionnaire-9	1
PSQ-20	Perceived Stress Questionnaire	1
SCID	Structured Clinical Interview for Mental Disorders	1
TAS	Toronto Alexithymia Scale	1
YRBSS	Youth Risk Behavior Surveillance System	1

**Fig. 3:**
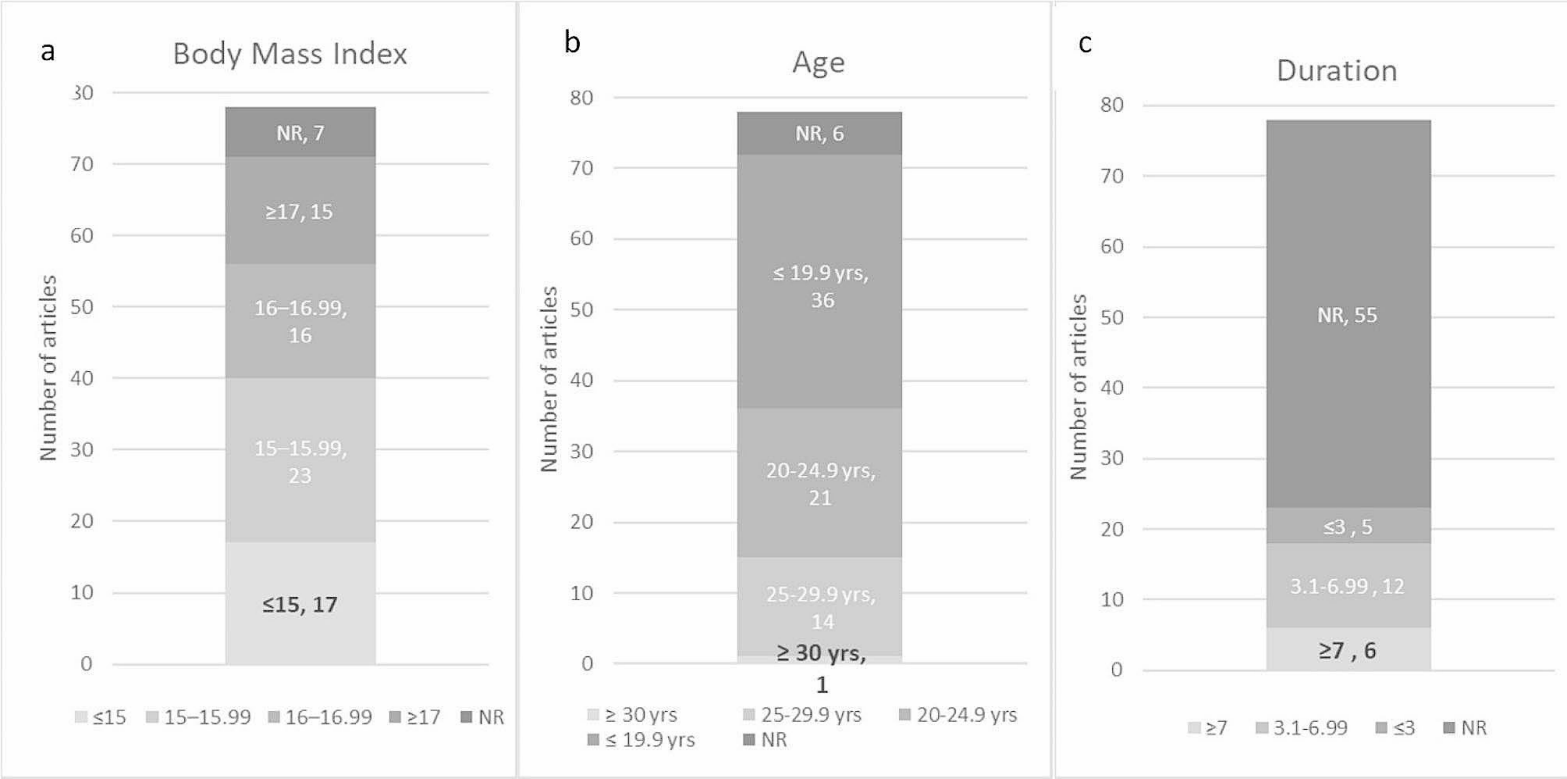
Number of articles in Table 1 representing the body mass index (BMI), age and duration subgroups indicated. NR = Not reported. A. BMI: 71 of the 78 articles reported BMI (kg/m^2^), 17 of those 71 had participant mean BMI ≤ 15; Age: 72 of the 78 articles reported age, of those 72, one had a mean participant age over 30 years; Duration: 23 of the 78 articles included duration, of those 23, 6 had participant mean illness duration of ≥ 7 years

Historically, the focus of AN research has been on teens and young adults. The current assessment found that, of the 71 studies in which the mean age was reported or could be calculated, the mean of the mean ages reported for study participants was 20.9 (4.26 SD) years. Furthermore, the reported mean age of study participants in 36 (51%) of the 71 studies was ≤19.9 years, 21 (30%) had a mean age of 20-24.9 years, 14 (20%) had a mean age of 25-29.9 years, and only one study had an overall group mean age of ≥ 30 years, although eight studies included individual groups with means ≥ 30 years. Figure 3 provides a summary of the BMI, age and duration findings discussed above.

Incidence rates for AN are reported to be ten times lower in males, although this is considered an underestimation due to underreporting and underdetection [2]. Only 16 (20%) of the 78 studies included male participants.

Based on the min/max and standard deviations of the mean provided for duration of illness and BMI, it was clear that many of the articles included subsets of individuals meeting the criteria noted herein for severe and enduring AN. However, as data for those specific individuals were often not delineated, it was not possible to determine how the study conclusions may have differed for said subgroups. For example, the mean duration of illness reported by Hernández et al. [75] for the AN restricting type (AN-R) subgroup was 4.03 (4.44 SD) years, indicating that at least some of the participants met the duration criteria.

Nevertheless, there were examples of results being assessed against some measures of severity, including duration. The Booij et al. study [76] AN-R group participant duration of illness was 54.9 (30 SD) months; range: 12–84. They specifically assessed methylation against the cumulative duration of illness and observed associations between duration and methylation levels at 142 probes. The mean duration of illness in the AN-R group in the Steiger et al. study [77] was 96.00 ± 98.91 (12–456) months. They also assessed duration and found an association between chronicity of illness and methylation status at 64 probes mapping to 55 genes.

Other authors evaluated genetic correlation with the severity of various psychological assessments including quality of life, depression, food behaviors, anxiety, and obsessiveness [75, 77–90]. For example, Acevedo and colleagues found a correlation between specific single nucleotide polymorphisms (SNPs) of the oxytocin receptor gene (*OXTR*), and increased severity of eating disorder symptoms in those with AN [78]. A polymorphism in the promotor region of the serotonin transporter gene (*5-HTTLPR*), previously associated with stress and depression [91], may impact depression and long-term outcomes in those with AN [79]. Research also suggests a possible correlation between specific haplotypes of the DHEA-producing enzyme cytochrome P450 CYP17A [81] and the C861 allele of the serotonin receptor 1Dβ gene (*HTR1B*) and severity of anxiety in those with AN.

An example of potential utility in assessing the severe and enduring AN phenotype and the need for larger studies and more funding is the 2022 study by Johansson et al. [50] evaluating polygenic association with AN severity and long-term outcomes. Here, the authors delineated severe and enduring AN criteria, including duration of illness, clinical impairment, BMI, and having undergone at least one previous treatment attempt. They also specified requirements for the AN subtype, thereby narrowing the population. The study, which included 2843 participants followed for up to 16 years (mean: 5.3 years), provided evidence supporting the possible clinical utility of PGSs for assessing eating disorder risk but also noted the need for larger studies and sample sizes to increase statistical power.

In summary, based on the literature reviewed, genetic studies of AN continue to focus largely, but not exclusively, on younger female participants with shorter durations of illness. These findings are not surprising given that the majority of those diagnosed with AN are female, the lack of clearly defined criteria for severe and enduring AN and the need for large numbers of participants to assess significance in genetics research.

## Discussion

Attempts to provide criteria for labeling those with severe mental illness as chronic or treatment-resistant need to be executed with care, as has been critically reviewed for illnesses such as schizophrenia and depression [92, 93]. Care should also be taken when defining criteria for severity of AN, which has a higher mortality rate than depression or schizophrenia [94]. However, not defining AN severity more clearly and not focusing on a more severe and enduring phenotype in research may decrease the likelihood of identifying the possible underlying biological etiology of AN. As noted by Wonderlich et al. [20] and responding commentaries by Dalle Grave [95], Wildes [96], and McIntosh [97], a lack of consensus and studies specifically targeting those with severe and enduring AN has resulted in patients being subjected to repetitive employment of largely ineffective treatment strategies resulting in a sense of hopelessness and shame and increasing the risk of suicide [98]. This review of the literature found that a duration of illness ≥7 years and an unsuccessful response to previous evidence-based treatment were the most common inclusion criteria employed, as were various measures of psychological and physical severity.

AN was once thought to be primarily caused by dysfunctional family dynamics and social and cultural pressures [99]. We now have evidence that genetics plays a significant role in its etiology. In recent years, there has been an evidence-based push to reconceptualize AN as a metabopsychiatric disorder [7]. Functional magnetic resonance imaging (fMRI) continues to provide data on the functioning of the brains of those with AN [100]. The use of large-scale GWAS and genome-wide methylation studies has been gradually revealing the interplay between genetics and environment in AN etiology and persistence, and genetic correlations with other psychiatric disorders [16, 101, 102]. These are all positive advances; however, as evidenced by the individuals included in these studies, female teens and young adults with shorter durations of illness appear to be the primary participants.

Historically, males have been underrepresented in AN research [103]. Until 2013, the DSM listed amenorrhea as a criterion for AN, thereby reinforcing the notion that AN affects only females [61]. According to the literature reviewed, males continue to be underrepresented in AN research.

The challenge of recruiting participants for inclusion in large-scale genetic studies of AN is significant. Of the indicated criteria, the most challenging for researchers to assess is the lack of response to prior evidence-based treatment. Most of the treatments described as evidence-based are not administered according to a defined protocol, making retrospective assessment nearly impossible. Furthermore, those with more severe symptoms of longer duration are often treated in a plethora of settings over many years.

For many of the publications, the data indicate that there were participants meeting the criteria defined in the first aim. However, as these individuals were not assessed as a group, it was not possible to determine whether outcomes for this subset may have differed from those with a less severe presentation. The purpose of the publications that either did not perform these assessments or did not report them in their studies was not to delineate this level of detail, so their absence is understandable. One of the reasons for this may be the small number of individuals meeting the criteria for severe and enduring AN, coupled with the need for a large enough “n” to provide any meaningful statistical assessment, which in turn points back to the need for larger studies and additional funding.

Nevertheless, several studies made concerted efforts to focus on a defined severe and enduring phenotype. For example, Kushima et al. [74] limited their study cohort to those reporting a lifetime lowest BMI < 15 kg/m^2^, with the median for included participants reported as 11.3 kg/m^2^, and a mean age of 37.9 years. The authors specifically stated that they focused on the “severe subgroup of patients because patients with severe symptoms or treatment-resistance are more likely to carry rare deleterious variants of large effect”, citing a schizophrenia study [104] as support.

The ultimate goal of AN research is to identify contributing factors to the manifestation and intractability of the disease and, in turn, develop superior evidence-based treatments tailored to the patient. Will next generation sequencing gene panels help in the diagnosis of AN [105]? Kushima et al. [74] suggested that rare copy number variants associated with neurodevelopmental disorders may correlate with more severe eating disorder subtypes. Is it possible to identify those at higher risk of developing severe and enduring illness earlier and in turn treat those patients based on their specific genetic and environmental circumstances instead of employing generic therapy that may work for most patients with eating disorders but is less effective for those in this cohort? Can artificial intelligence be employed to better identify risk in individuals with AN [106]? Will we one day regularly employ genetic testing and pharmacogenetics in treating mental illness, including AN [107, 108]? Several international projects, including ANGI and the Comprehensive Risk Evaluation for Anorexia Nervosa in Twins (CREAT) are attempting to answer these questions and many more [109, 110]. Although these projects do not focus specifically on the severe and enduring phenotype, the availability of in-depth participant health and demographic information paired with genetic analysis should allow for studies of these subsets.

The criteria for evaluating the severity and intractability of AN are evolving, as is the understanding of the disorder. The purpose of a scoping review is to map the literature on an evolving topic and to identify gaps. As such, unlike a systematic review, this review does not attempt to assess the quality of the research conducted, but rather the inclusiveness of study participants. The authors do not attempt to define the severe and enduring phenotype or suggest how the research community should create consensus on the definition. However, by assessing the current literature, we highlight the gaps between the intent to focus on those with severe and enduring AN and the inclusion of this group in published research.

## Conclusion and future directions

In conclusion, this review provides an overview of the currently used criteria employed by the research community to define the severity of AN and assesses the last decade of genetics research for the inclusion of study participants meeting these criteria. We found that the following combination of assessments and criteria was used most often in the literature to define AN severity and intractability:

1. Illness duration of ≥ 7 years.
2. lack of positive response to at least two previous evidence-based treatments.
3. A BMI meeting the DSM-5 criteria for extreme AN.
4. An assessment of psychological and/or behavioral severity indicating a significant impact on quality of life.

We also found, especially in recent years, that there has been an attempt to better define severe and enduring AN in hopes of identifying patients, tailoring treatment, and improving outcomes. However, although a small subset of genetic studies reviewed specifically attempted to focus on a severe and enduring phenotype, there was a lack of aligned defining criteria. Furthermore, there is a continued focus on younger females with shorter disease durations.

Those with AN are often stigmatized, and their shame is amplified by the perception that AN is voluntary or even a lifestyle choice [111–113]. Those with severe and long-lasting illness are less likely to respond to currently available treatment modalities and have higher levels of mortality [20]. However, they also represent a subgroup of individuals for which genetic findings may be especially helpful [74]. Therefore, it is suggested that future genetics studies make a concerted effort to include older participants, those with longer illness durations, and those whose quality of life is most significantly impacted. It is also critically important that more objective, empirically based techniques, such as biomarker and brain structure and function analysis, be developed to more definitively classify the severe and enduring phenotype, which to this point has primarily been categorized through subjective means [32, 60, 96, 114]. There has been considerable effort in recent years to expand the definition of AN in hopes of being more inclusive and identifying those who may benefit from treatment. However, although expansion has increased the sample size for genetic studies, it could be that focusing on those with longer-lasting and more severe symptomology, even though this is a much smaller group of those with AN, would provide a better chance of identifying the genetic etiology of the disorder. Recent advances have left us far better equipped to make significant progress in developing evidence-based treatments for those with severe and enduring AN. However, these advances require the inclusion of this subgroup in both research and practice.

### Limitations

One limitation of the current review is that due to the wide range of similar terminology used to refer to a severe and enduring AN phenotype in the published literature, the searches performed may have left out pertinent articles and viewpoints. Furthermore, although comprehensive for the three electronic databases, the literature search did not include gray literature; thus, information from sources such as dissertations may have been missed.

### Electronic supplementary material

Below is the link to the electronic supplementary material.

Supplementary Material 1

Supplementary Material 2

## Data Availability

No datasets were generated or analysed during the current study.
